# Factors Related to Anxiety, Depressive Symptoms and Quality of Life in Breast Cancer

**DOI:** 10.3390/ijerph19063547

**Published:** 2022-03-16

**Authors:** Macarena C. Cáceres, Marta Nadal-Delgado, Casimiro López-Jurado, Demetrio Pérez-Civantos, Jorge Guerrero-Martín, Noelia Durán-Gómez

**Affiliations:** 1 Departamento de Enfermería, Facultad de Medicina y Ciencias de la Salud, Universidad de Extremadura, 06006 Badajoz, Spain; casimirolj@unex.es (C.L.-J.); jorguerr@unex.es (J.G.-M.); nduran@unex.es (N.D.-G.); 2 Hospital Universitario de Badajoz, 06006 Badajoz, Spain; mnadal.aoex@yahoo.es; 3 Facultad de Medicina y Ciencias de la Salud, Universidad de Extremadura Hospital Universitario de Badajoz, 06006 Badajoz, Spain; dperciv@unex.es

**Keywords:** breast neoplasm, quality of life, depressive symptoms, anxiety

## Abstract

Breast cancer (BC) is a major public health problem internationally. Although illness survival rates have improved, patients usually suffer multiple symptoms, both physical and psychological, which can affect their quality of life (QoL). The main aim of this study was to evaluate depressive symptoms, anxiety and the QoL of people with BC. An observational, cross-sectional study was carried out at Badajoz University Hospital (Spain). A total of 200 women with BC were included. EORTC QLQ-C30 and QLQ-BR23 questionnaires were used to assess QoL. Patients were screened for depressive symptoms using the Beck Depression Inventory (BDI) and for state anxiety and trait anxiety using the State Anxiety Inventory (STAI). Thirty-eight percent of the patients in the sample had moderate to severe anxiety, which was related to the time of diagnosis, advanced stage of illness and surgical treatment. We found that 28% of patients had depressive symptoms, related mainly with time of diagnosis, adjuvant therapy and number of cycles of chemotherapy (CT). Patients with the longest time since diagnosis, in stage III, and in treatment with CT, especially those with the greatest number of cycles, had the worst scores in QoL. We found a positive association between depressive symptoms and anxiety with QoL in patients with BC.

## 1. Introduction

Breast cancer (BC) is a major public health problem internationally. It was the most commonly diagnosed cancer in 2020, with an estimated 2.3 million new cases, representing 11.7% of all cancer cases [1]. At the time of writing, it had been estimated that in 2021 the number of people diagnosed with BC in Spain would be 33,375 [2], without taking into account the possible influence of the COVID-19 pandemic in Spain and the rest of the world. Data from both GLOBOCAN and REDECAN are based on data prior to the COVID-19 pandemic, which may affect their reliability [3].

Although illness survival rates have improved (in Spain survival at 5 years is 85.5%) [3], patients usually report having multiple symptoms, both physical and psychological, which can affect their quality of life (QoL) [4]. BC patients may experience psychological problems related to diagnosis, effects of treatment and end of life [5,6]. It has been demonstrated that BC patients have high levels of anxiety, depressive symptoms and lower QoL [7,8,9,10]. These alterations in mood in oncological patients can result, furthermore, in non-compliance with treatment, longer hospitalization, wrong prognosis and increased mortality [11,12,13].

Several studies have assessed the prevalence of anxiety in BC; taking into account the different methodologies used in these studies, the rates range from 33 to 60% [14,15,16,17]. Depressive symptoms are also an important psychological consequence of BC. Prevalence rates of depression in people with BC range between 25 and 66.6% [5,15,18,19].

Numerous studies have analyzed the relation between the group of symptoms found in BC and QoL [18,20,21], but great heterogeneity exists in the types of symptom clusters identified in the different studies reviewed. This could be related to the fact that patients are subjected to different treatment regimens, in addition to the different methodologies used to identify symptoms. So et al. (2021) propose separately examining the symptoms that occur in BC [8].

Focusing on the analysis of QoL in women with BC, we found many studies conducted at different stages of the disease or with different types of treatment [22,23,24,25]. Other authors have focused on the QoL of women who received and did not receive all recommended adjuvant treatments for BC [26,27]. Some studies have highlighted the importance of knowing the QoL of women with BC belonging to different cultures [28,29,30,31]. Specifically, the relationship between psychoneurological symptoms (anxiety and depression) and QoL has been previously studied, finding that patients with BC experience anxiety and depression, among other symptoms, and loss of QoL during treatment; however, these symptoms are often overlooked [9]. In the Spanish population, only a few studies have analyzed QoL in women with BC [30,32], but they do not analyze its relationship with psychoneurological symptoms, or are limited to women of certain climacteric groups (premenopausal).

There are a lack of studies in this field that analyze the relationship between depressive symptoms, anxiety and their impact on QoL in Spanish women with BC undergoing different regimens of treatment, alone or in combinations. It is of great relevance that health professionals are aware of the burden of these symptoms, and are able to identify those women who are most likely to suffer from them, given the importance of adhering to treatment and the impact they can have on QoL.

The main aim of our study was to evaluate depressive symptoms, anxiety and QoL of a Spanish population with BC receiving different types of oncological treatment.

## 2. Materials and Methods

### 2.1. Sample and Settings

We performed an observational, cross-sectional, non-probability study between January 2017 and February 2020 at Badajoz University Hospital (Spain). Women diagnosed with BC receiving oncological treatment were included. All fulfilled the inclusion and exclusion criteria. Exclusion criteria were: being a minor; being over 85 years; not being a patient of Badajoz University Hospital; not signing the informed consent form; having neurological or cognitive impairment which would impede carrying out the assessment; having previously received treatment for another type of primary cancer; having a diagnostic record of comorbidity associated with depression, anxiety and/or cognitive impairment; having linguistic or communicative barriers; having a previously diagnosed psychiatric disorder; being under psychopharmacological and/or psychotherapeutic treatment.

### 2.2. Procedure

Identification of the cases was carried out by the Medical Oncological Service of Badajoz University Hospital. Inclusion and exclusion criteria were then revised, and the programmed activity for each patient was reviewed with a view to their participation in the study when they attended the hospital for their next appointment. Once the informed consent form was signed, a trained researcher conducted the clinical interview. After the first part of the interview, each participant was given the study questionnaires and they filled them in on their own. All documents were completed face-to-face in the Medical Oncological Service with the support of a trained researcher.

Permission was obtained from the Ethics in Clinical Investigation Committee of Badajoz. Confidentiality of the information was maintained at all times in accordance with current legislation (Spanish Organic Law 3/2018, of 5 December, on Protection of Personal Data and Guarantee of Digital Rights).

### 2.3. Methods and Variables

Clinical interview: a clinical interview was used to assess self-reported sociodemographic data, and clinical and psychological variables of the patient.

Clinical history: the clinical history was used to assess characteristics of the tumor, pathological anatomy and therapeutic management variables.

Mood and QoL measurements: the patients were screened for depressive symptoms using the Beck Depression Inventory (BDI II) [33] and for anxiety using the State Trait Anxiety Inventory [34]. Patients completed the EORTC QLQ-C30 (3.0) [35] and the QLQ-BR23 [36] questionnaires, which had been translated into Spanish [37] and validated for use in Spain [38,39].

EORTC QLQ-C30: is a 30-item questionnaire assessing function and symptoms that impact QoL in people with cancer. It is subdivided into three scales: global health status and QoL (GHS); functional scales relating to physical functioning (PF), role functioning (RF), emotional functioning (EF), cognitive functioning (CF) and social functioning (SF); and symptom scales relating to fatigue (FA), nausea and vomiting (NV), pain (PA), dyspnea (DY), insomnia (SL), appetite loss (AP), constipation (CO), diarrhea (DI) and financial difficulties (FI). A high score for a functional scale represents a high/healthy level of functioning, a high score for the global health status/QoL represents a high QoL, but a high score for a symptom scale/item represents a high level of symptomatology/problems [40].

The QLQ-BR23 questionnaire is a QLQ-C30 supplemental questionnaire created specifically for people with BC. It consists of 23 questions, which are subdivided into two scales: the functional scales, composed of body image (BRBI), sexual functioning (BRSEF), sexual enjoyment (BRSEE) and future perspective (BRFU); and the symptom scales, consisting of the subscales systemic therapy side effects (BRST), breast symptoms (BRBS), arm symptoms (BRAS) and upset by hair loss (BRHL). The scoring approach for the QLQ-BR23 is identical in principle to that for the function and symptom scales/single items of the QLQ-C30 [41].

The BDI-II, adapted to Spanish language [42] assesses the symptoms of depression in the individual in the previous two weeks. The standard cut-off score is 14 and the presence and degree of symptoms of depression can be detected. Scores 0–13 indicate no or minimal depression, scores 14–19 indicate mild depression, scores 20–28 indicate moderate depression, and scores 29–63 indicate severe depression [33].

The State Trait Anxiety Inventory, adapted to Spanish language [43] includes separate scales of auto-evaluation which measure two concepts of anxiety: state anxiety (S/A) and trait anxiety (T/A). Each of the two STAI scales consist of 20 items: one part of them is written in positive terms, and the other in negative terms. Scores ≤ 21 indicate mild anxiety (percentile 50), scores 22–31 indicate moderate anxiety (percentile 75) and scores 32–60 indicate severe anxiety (percentile 99).

#### Statistical Analysis

The socio-demographic and clinical characteristics of the total number of enrolled women were analyzed with descriptive statistics: T-student, ANOVA and Chi Square tests were used as required. The correlation between the quantitative variables was calculated by means of Pearson’s correlation coefficient. Specifically, time since diagnosis was considered an independent variable and its effect on the other variables was analyzed by lineal regression.

All statistical analyses were performed using IBM Corp. Released 2013. IBM SPSS Statistics for Windows, Version 22.0. Armonk, NY: IBM Corp. For all analyses, the α-level was set at *p* ≤ 0.05.

## 3. Results

Two hundred women participated in the present study, with an average age of 53.05 ± 10.71 years, an average time since diagnosis of BC of 22.79 ± 42.06 months, and an average number of chemotherapy (CT) cycles of 6.04 ± 8.38. A description of the sociodemographic and clinical variables is presented in Table 1.

**Figure 1:**
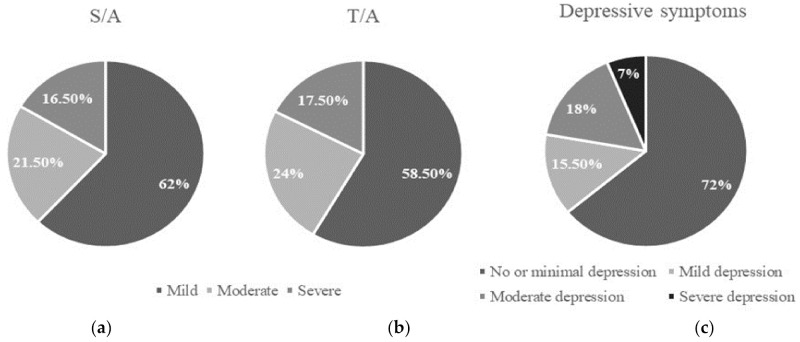
(**a**) Percentage of patients with mild, moderate or severe S/A; (**b**) percentage of patients with mild, moderate or severe T/A; (**c**) percentage of patients with no or minimal, mild, moderate or severe depression measured with BDI.

The average scores for anxiety were 19.06 ± 12.18 for S/A and 22.52 ± 9.78 for T/A. Twenty-eight percent (n = 56) of the women had clinically relevant depressive symptoms (cut-off point ≥ 14). The average of the scores from the BDI questionnaire was 10.66 ± 8.27. Figure 1 shows the scores on anxiety and depressive symptoms categorized by levels.

When the scores obtained from the anxiety and depression questionnaires were compared, a correlation between the two dimensions of STAI (S/A and T/A: r = 0.583, *p* < 0.001) was found, and also between both of these and depressive symptoms (S/A and BDI: r = 0.573, *p* < 0.001; T/A and BDI: r = 0.550, *p* < 0.001).

Regarding the relation between the socio-demographic variables and the anxiety and depression scales, we found a relation between the presence of depressive symptoms and married status (χ^2^ = 11,288; *p* = 0.024), concretely in the BDI category of moderate depression (χ^2^ = 24,915; *p* = 0.015).

### 3.1. Anxiety, Depressive Symptoms, and Other Factors

Time passed since diagnosis related significantly with S/A (r = 0.139; *p* = 0.040) and depressive symptoms (r = 0.169; *p* = 0.017), in such a way that the longer the time since diagnosis, the higher the prevalence these symptoms had. Specifically, we applied a one-way ANOVA test and found a relation between time passed since diagnosis and severe anxiety (percentile 99) (*p* = 0.026). Additionally, time since diagnosis was related with depressive symptom scores corresponding to moderate depression (scores 20–28) (*p* = 0.040). For the current situation, it was found that in patients who were having check-ups there were high scores for S/A (severe anxiety) (χ^2^ = 14,908; *p* = 0.005) which, in turn, was related to the time elapsed since the diagnosis, which characterizes this subgroup of patients under check-ups.

S/A scores results were significantly associated to stage III with respect to stage 0 (*p* = 0.011) (Table 2).

We analyzed the differences between the treatment combinations as a whole and separately (CT only, surgery only, CT + surgery and other combinations), highlighting the differences described as follows (Table 3).

On studying the relation between mood and number of CT cycles, it was found that the highest scores for depressive symptoms corresponded to the highest number of cycles received (r = 0.153; *p* = 0.031), specifically for the level of moderate depression (*p* = 0.036). Similarly, having received ≥4 CT cycles was correlated with higher scores for depressive symptoms (*p* = 0.009).

For type of treatment received, the surgery variable was related with S/A (χ^2^ = 11,878, *p* = 0.003). It was observed that 93.9% (n = 31) of patients with severe anxiety and 81.5% (n = 101) with mild anxiety had undergone surgery with respect to patients without surgical treatment. Additionally, a relation was also found with the type of surgery. Of the total number of women with severe anxiety, only 4.9% were patients without surgical treatment, compared to 16.3% that were mastectomized (χ^2^ = 12,491, *p* = 0.014).

### 3.2. Quality of Life

The scores for the different scales of QoL corresponding to the EORTC QLQ-C30 and EORTC QLQ-BR23 are shown in Table 4.

On analyzing the relations on the QoL scales with the clinical variables, differences were obtained concerning current situations, with worse scores on the symptom scales of PA, FI and BRAS, and on the functional scales of BRBI, in women who were having check-ups compared to those commencing treatment ($\bar{X}$ = 43.86 ± 32.68, *p* = 0.027; $\bar{X}$ = 31.56 ± 34.18, *p* = 0.042; $\bar{X}$ = 29.21 ± 28.24, *p* = 0.048; $\bar{X}$ = 63.18 ± 32.78, *p* = 0.031, respectively).

The GHS, BRBI and CO scales depend directly on time passed since diagnosis, with the level of QoL being higher with the more time passed since diagnosis (r = −0.153 *p* = 0.031, r = −0.165 *p* = 0.020 and r = 0.151 *p* = 0.033, respectively).

Women who had surgically and/or drug-induced menopause prior to diagnosis had worse scores on the GHS scale with respect to natural menopause, analyzed by one-way ANOVA followed by the Bonferroni post-hoc method ($\bar{X}$ = 34.01 ± 9.43, *p* = 0.003;$\;\bar{X}$ = 23.02 ± 2.97, *p* = 0.009; $\bar{X}$ = 23.20 ± 2.54, respectively).

With regard to illness stage, women in stage III showed worse SF ($\bar{X}$ = 29.15 ± 5.07, *p* = 0.028) and greater FA ($\bar{X}$ = 32.23 ± 5.61, *p* = 0.039) with respect to stage I ($\bar{X}$ = 25.75 ± 4.95; $\bar{X}$ = 25.85 ± 3.28, respectively). Women in stage I had better values for BRBI ($\bar{X}$ = 21.17 ± 2.68, *p* = 0.021) than stage III ($\bar{X}$ = 32.97 ± 5.74).

Scores for women who had surgery treatment were worse for symptoms CO (surgery group: $\bar{X}$ = 23.67 ± 31.89, *p* = 0.028; without surgery group: $\bar{X}$ = 13.01 ± 25.69) and BRBS (surgery group: $\bar{X}$ = 19.95 ± 22.20, *p* = 0.013; non surgery group: $\bar{X}$ = 11.58 ± 17.47).

Patients in CT treatment displayed worse scores on the functional scales of PF, BRBI and BRSEE (*p* = 0.029, *p* = 0.004 and *p* = 0.048, respectively) and on the symptom scales of FA (*p* = 0.033), NV (*p* = 0.002), DY (*p* = 0.001), SL (*p* = 0.039), CO (*p* = 0.027), DI (*p* = 0.013), BRST (*p* = 0.001) and BRHL (*p* = 0.006). The mean levels of scores for CT treatment and without it were: PF ($\bar{X}$_1_ = 77.06 ± 22.00; $\bar{X}$_2_ = 86.18 ± 19.29), BRBI ($\bar{X}$_1_ = 74.84 ± 27.71; $\bar{X}$_2_ = 90.48 ± 14.11), BRSEE $(\bar{X}$_1_ = 75.39 ± 35.44; $\bar{X}$_2_ = 89.29 ± 25.74), FA ($\bar{X}$_1_ = 32.89 ± 27.92; $\bar{X}$_2_ = 21.01 ± 21.65), NV ($\bar{X}$_1_ = 7.97 ± 18.22; $\bar{X}$_2_ = 1.89 ± 7.93), DY ($\bar{X}$_1_ = 11.41 ± 26.56; $\bar{X}$_2_ = 1.19 ± 6.29), SL ($\bar{X}$_1_ = 37.76 ± 36.93; $\bar{X}$_2_ = 23.81 ± 31.23), CO ($\bar{X}$_1_ = 23.43 ± 31.82; $\bar{X}$_2_ = 9.52 ± 21.95), DI ($\bar{X}$_1_ = 10.25 ± 21.71; $\bar{X}$_2_ = 1.79 ± 6.93), BRST ($\bar{X}$_1_ = 28.68 ± 20.63; $\bar{X}$_2_ = 14.16 ± 12.90) and BRHL ($\bar{X}$_1_ = 23.30 ± 36.77; $\bar{X}$_2_ = 3.57 ± 13.87).

For the variable number of CT cycles, a close relation was found with the PF and BRBI scales (r = −0.201 *p* = 0.004 and r = −0.244 *p* = 0.001). Additionally, a direct correlation was found with symptoms of NV (r = 0.221 *p* = 0.002), CO (r = 0.170 *p* = 0.016) and BRAS (r = 0.166 *p* = 0.019).

Women with ≥4 CT cycles had worse scores on the GHS scales, PF and BRBI (*p* = 0.004, *p* = 0.001 and *p* = 0.001 respectively), and on the symptom scales with FA (*p* = 0.015), NV (*p* = 0.048), PA (*p* = 0.008), CO (*p* = 0.010), BRST (*p* = 0.002) and BRAS (*p* = 0.001). The mean levels of scores for ≥4 CT or <4 CT cycles groups were: GHS ($\bar{X}$_1_ = 56.22 ± 25.38; $\bar{X}$_2_ = 66.88 ± 24.22), PF ($\bar{X}$_1_ = 71.54 ± 21.30; $\bar{X}$_2_ = 82.09 ± 21.27), BRBI ($\bar{X}$_1_ = 65.98 ± 30.56; $\bar{X}$_2_ = 83.11 ± 22.32), FA ($\bar{X}$_1_ = 37.53 ± 28.01; $\bar{X}$_2_ = 27.75 ± 26.53), NV ($\bar{X}$_1_ = 10.32 ± 21.14; $\bar{X}$_2_ = 5.34 ± 14.42), PA ($\bar{X}$_1_ = 36.15 ± 31.87; $\bar{X}$_2_ = 24.78 ± 26.99), CO ($\bar{X}$_1_ = 29.10 ± 33.77; $\bar{X}$_2_ = 17.29 ± 28.59), BRST ($\bar{X}$_1_ = 32.57 ± 19.82; $\bar{X}$_2_ = 23.39 ± 19.93) and BRAS ($\bar{X}$_1_ = 26.43 ± 26.85; $\bar{X}$_2_ = 13.50 ± 19.35).

### 3.3. Anxiety, Depressive Symptoms and QoL

All the items on the EORTC QLQ-C30 scale were significantly related with the scores for S/A (except NV, DY and DI) and T/A (except NV) (Table 5). With regard to QLQ BR23, all the items on the questionnaire were related with S/A, T/A and BDI, except BRSEF and BRSEE (Table 6).

## 4. Discussion

As far as we know, the present study is the first to attempt to evaluate depressive symptoms, anxiety and QoL of people with BC in different types of treatment in a Spanish population. Therefore, we evaluated the prevalence of anxiety and depressive symptoms in women with BC and the relation of these to QoL. In our study, 28% of the number of women enrolled with BC presented symptoms of depressive disorders. Moderate to serious levels of S/A were found in 38% of women, and of T/A in 41.5%. Notwithstanding methodological differences with other studies on prevalence of depression and anxiety in BC patients, our results were similar to those published in the literature [17,44].

We found that, in our patients, the longer the time passed since diagnosis, the higher the levels of S/A and depressive symptoms. We also found a significant relation between severe anxiety and check-up situation. Some studies have reported that when cancer is initially diagnosed, anxiety increases in an natural way, then diminishes with time as the patient adapts to the illness, but that it can increase again at a later point if the symptoms become more serious [45]. The time of diagnosis, the course of CT treatment and the months following the end of the treatment are times of bad adaptation to the transition and of fluctuating anxiety [46]. Other studies, however, have found that patients diagnosed with BC felt anxious and depressed, but this tended not to change significantly during the therapeutic procedures [9], and it was found that these symptoms persisted for long periods [47]. It has been suggested that psychological symptoms are a result of both the cancer itself and the harmful effects of its treatment [8].

We found higher levels of anxiety in women at advanced stages, specifically stage III. Other studies also relate levels of anxiety or depression with advanced stages of the illness [48,49,50]. This could be explained by the presence of symptoms related to cancer such as fatigue, pain, nausea, etc., as well as uncertainty and fear of metastasis.

Treatment for BC is another anxiety- and depression-inducing factor [51,52,53].

With regard to type of treatment received by patients, we found that the women who had undergone surgery had more anxiety than those who have received other treatments, such as CT. Several studies have reported higher anxiety levels related to surgery versus other treatments, specifically among mastectomized women [42], and we were able to confirm this fact. This research found a higher level of anxiety among the mastectomy group, suggesting that women were more likely to suffer from emotional turmoil after undergoing mastectomy that could aggravate coping with BC and attendant treatment. We can suggest that the impact of breast surgery type as a physical factor on patients’ psychological adjustment may be significant, and thus surgery is a physical factor that contributes to anxiety. This is not consistent with previous research, since when comparing treatment modalities, women receiving radiotherapy or CT tend to exhibit a higher anxiety score over time compared to those receiving surgery [54]. Level of anxiety has also been reported to be higher in patients undergoing CT as compared to radiotherapy, an aspect that did not find. We did not observe a relationship between depressive symptoms and the surgical treatment modality; however, Salibasic and Delibegovik have published that, although depression affects all patients with BC, it is especially present in patients who have undergone a radical surgical procedure [24].

It has been shown that CT can aggravate emotional symptoms, among other things, as a result of the changes in body image induced by the treatment [55], with social activity limitations or poor functional status [51]. A positive relation has been found between the number of CT cycles and depressive symptoms. Whishenant et al. (2020) related CT cycles with depressive symptoms, finding that 60% of women had a depressed mood at cycle 2 CT, and 53% at cycle 3 [5]. Zhang et al. (2018) also found a relation between CT and anxiety and depression, which was highest in the third cycle [46]. Furthermore, in our study we related the adjuvant therapy variable based on standard dose polychemotherapy regimens with the presence of depressive symptoms, so we can confirm not only the relationship of CT and surgery with the presence of depressive symptoms separately, but also that the association of both treatment modalities enhances the appearance and impact of these symptoms. Other authors have found a higher risk of depressive disorders in patients with BC at the age of 40–59, this risk increasing for those patients who had undergone adjuvant therapies including CT, radiotherapy, tamoxifen, third-generation aromatase inhibitors, or trastuzumab [56].

The global health status of the whole sample shows normal results (63.1 ± 25.1). All the scores in the functional scales were high, except for the emotional scale, which coincides with another study on the Spanish population [57]. With regard to the symptom scales, we observed that the symptoms which most affected QoL were insomnia, fatigue, pain and constipation, coinciding with the literature [4,58]. From a functional point of view, future perspective seemed to be the most affected scale. Different factors, both psychological (higher levels of mental stress or depression), and social and physical, have been reported as possible determinants of future perspectives [30,59].

Our results revealed worse QoL (pain, financial difficulties, body image and arm symptoms) in women who were receiving initial treatment, compared to those who were in remission or having check-ups. Worse scores were also found in QoL (global health status, constipation and body image) in women for whom a long time had passed since diagnosis. For some authors, worse levels of QoL were also found with longer time since diagnosis [28], although other prospective follow-up studies have reported that QoL improves sometime after treatment [57,60]. Furthermore, we found worse scores in advanced stages, such as stage III. There is some evidence that advanced stage BC disease is associated with poorer QoL [4].

Worse results in QoL were observed in patients undergoing CT, and with those who had the highest number of cycles. Other authors found that CT in itself can affect QoL of women with BC [27], and differences have been noted between QoL and number of cycles, QOL being lowest at the third CT cycle and highest at the first [22].

We found differences between QoL related to anxiety and depressive symptoms in all the items on the EORTC QLQ-C30 questionnaire (except nausea and vomiting, dyspnea and diarrhea for S/A and nausea and vomiting for T/A) and on the QLQ-BR23 questionnaire (except sexual functioning and sexual enjoyment). It has been widely demonstrated that both anxiety [55] and depressive symptoms have a significant impact on the QoL of individuals with BC [9,61]. It would seem, however, that emotional symptoms of anxiety and depression diminish after treatment, and that QoL improves in patients with BC [52,57].

Our study is not without limitations. A longitudinal design, in which variables are measured before, during and after treatment, would have identified the factors which can affect QoL with greater precision. Every effort was made, however, to include the highest possible number of patients from our hospital during the recruiting period with the initial objective of demonstrating the existence of a relationship between the variables studied and QoL. Neither have we studied the fear of recurrence [62]—which is one of the most prevalent, persistent, and disruptive problems for the cancer survivors group—or protective factors such as social support [14]. In this sense, this aspect and those mentioned above will be analyzed in future studies within our line of research in BC patients.

## 5. Conclusions

The results corroborate our hypothesis that women with BC undergoing treatment presented anxiety and depressive symptoms which affect their QoL. Breast surgery affects the psychological outcomes of women with BC by increasing anxiety levels; moreover, adjuvant therapy based on standard dose polychemotherapy regimens increased the presence of depressive symptoms compared to other treatments. Time since diagnosis, stage of illness and number of CT cycles were the variables which exerted the greatest influence on the emotional state of these patients and their QoL.

This investigation found evidence to conclude that emotional symptoms are a prominent issue surrounding treatments for BC when examining the main treatment modalities. Despite this similarity, each treatment modality contributed to this symptomatology in different ways, but with a very clear and determinant implication on QoL. BC patients have multiple physical, emotional and psychological needs, which cannot be satisfied within current health systems. It is important to raise awareness among health professionals of the problems and needs of BC survivors, and to provide resources for them based on research evidence [63]. With the prevalence, intensity and correlated factors of identified emotional symptoms, it will be worthwhile for further research to investigate interventions that could help alleviate emotional symptoms among this group of patients, in order to help them attain a better level of QoL.

## Figures and Tables

**Table 1:** Social, demographic and clinical characteristics of the patients included in the study (mean age ± standard deviation).

Variable	Categories	N (%)/M ± SD
Age		53.05 ± 10.71
Marital status	Married	145 (72.5)
	Single	21 (10.5)
	Divorced	15 (7.5)
	Widowed	19 (9.5)
Education level	No studies	15 (7.5)
	Elementary school	59 (34)
	Middle school	27 (13.5)
	High school	44 (22.0)
	Higher education	55 (27.5)
Employment situation	Currently in employment	22 (11)
	Temporary sick leave	80 (40)
	Permanent sick leave	16 (8)
	Unemployed	50 (25)
	Retired	32 (16)
Tumor staging	0	10 (5)
	I	62 (31)
	II	68 (34)
	III	33 (16.5)
	IV	27 (13.5)
Grade	1	34 (17)
	2	57 (28.5)
	3	109 (54.5)
Molecular subtype	Luminal A/Luminal B HER2 negative-like	106 (53.0)
	Luminal B HER2 positive-like/HER2-type	73 (36.5)
	Triple negative	21 (10.5)
Current situation	Initial treatment	147 (73.5)
	Relapse	34 (17)
	Check-ups	19 (9.5)
Therapy	Neoadjuvant	22 (11)
	Adjuvant	178 (89)
Menopause	Natural	83 (41.5)
	Drug-induced menopause	60 (30)
	Intervention-induced menopause	13 (6.5)
	Reproductive stage	44 (22)
Treatment	Surgery	159 (79.5)
	Chemotherapy	172 (76)
	Radiotherapy	90 (45)
	Hormonotherapy	75 (37.5)
	Immunotherapy	42 (21)
Treatment combinations	Chemotherapy	23 (11.5)
	Surgery	8 (4)
	Surgery and chemotherapy	35 (17.5)
	Surgery, chemotherapy and radiotherapy	21 (10.5)
	Surgery, chemotherapy, radiotherapy and hormonotherapy	30 (15)
	Surgery, chemotherapy, radiotherapy, hormonotherapy and immunotherapy	12 (6)
	Other combinations	71 (35.5)
Surgical treatment	Conservative surgery	110 (55)
	Uni- or bilateral mastectomy	49 (24.5)
	Without surgical treatment	41 (20.5)
Chemotherapy cycles	Chemotherapy cycles < 4	72 (41.9)
	Chemotherapy cycles ≥ 4	100 (58.1)

**Table 2:** Relation between scores on the STAI and BDI questionnaires and tumor staging.

	S/A M ± SD	T/A M ± SD	BDI M ± SD
Stage 0	11.00 ± 1.32 ^a^	19.20 ± 11.02	9.50 ± 7.15
Stage I	16.24 ± 11.19 ^a,b^	20.69 ± 9.72	9.95 ± 8.72
Stage II	20.68 ± 11.51 ^a,b^	23.82 ± 9.76	9.63 ± 5.92
Stage III	23.30 ± 13.23 ^b^	24.94 ± 8.89	13.24 ± 10.55
Stage IV	19.22 ± 13.26 ^a,b^	21.67 ± 10.03	12.11 ± 9.28
	*p* = 0.011	*p* = 0.149	*p* = 0.220

^a,b^ Indicate that there is a significant difference between the two groups according to Tukey’s method only if they do not have any letters in common.

**Table 3:** Relation between scores on the STAI and BDI questionnaires and therapeutic management.

			S/A		T/A		BDI	
			M ± SD	*p*	M ± SD	*p*	M ± SD	*p*
Treatment	Surgery	No	18.88 ± 10.50	0.917	23.24 ± 10.72	0.594	10.29 ± 9.41	0.754
		Yes	19.10 ± 12.62		22.33 ± 9.55		10.75 ± 7.99	
	Radiotherapy	No	18.03 ± 10.82	0.188	21.68 ± 9.32	0.184	10.09 ± 8.71	0.288
		Yes	20.31 ± 13.63		23.53 ± 10.27		11.34 ± 7.71	
	Hormonotherapy	No	17.87 ± 11.07	0.076	22.53 ± 9.57	0.981	10.19 ± 8.18	0.308
		Yes	21.03 ± 13.70		22.49 ± 10.18		11.43 ± 8.43	
	Immunotherapy	No	18.47 ± 11.93	0.192	22.39 ± 9.90	0.719	10.35 ± 8.25	0.310
		Yes	21.24 ± 13.03		23.00 ± 9.43		11.81 ± 8.37	
Surgical treatment	Surgical treatment: mastectomy		17.31 ± 12.32	0.593	21.67 ± 10.98	0.661	10.29 ± 7.83	0.194
	Surgical treatment: conservative surgery		19.90 ± 12.71		22.62 ± 8.88		10.95 ± 8.09	
	Without surgery		18.88 ± 10.50		23.24 ± 10.72		10.29 ± 9.41	
Chemotherapy cycles	Chemotherapy cycles < 4		18.33 ± 11.47	0.260	21.73 ± 9.81	0.126	9.53 ± 7.35	0.009
	Chemotherapy cycles ≥ 4		20.37 ± 13.37		23.94 ± 9.64		12.70 ± 9.46	
			**r**	***p***	**r**	***p***	**r**	***p***
	Number of chemotherapy cycles		0.082	0.247	0.028	0.692	0.153	0.031

**Table 4:** Mean values ± standard deviation of the functional and symptoms scales of EORTC QLQ-C30 and EORTC QLQ-BR23.

EORTC QLQ-C30	N = 200
	Mean ± SD
Global health status/QoL	63.1 ± 25.1
Functional scales	
Physical functioning (PF)	78.34 ± 21.83
Role functioning (RF)	77.18 ± 28.12
Emotional functioning (EF)	72.56 ± 23.78
Cognitive functioning (CF)	80.57 ± 27.26
Social functioning (SF)	76.57 ± 26.79
Symptom scales/items	
Fatigue (FA)	31.23 ± 27.40
Nausea and vomiting (NV)	7.11 ± 17.22
Pain (PA)	28.81 ± 29.25
Dyspnea (DY)	9.98 ± 24.99
Insomnia (SL)	35.81 ± 36.44
Appetite loss (AP)	12.16 ± 23.91
Constipation (CO)	21.49 ± 30.97
Diarrhea (DI)	8.82 ± 20.44
Financial difficulties (FI)	16.82 ± 28.93
**EORTC QLQ-BR23**	**N = 200**
	**Mean ± SD**
Functional scales	
Body image (BRBI)	77.03 ± 26.76
Sexual functioning (BRSEF)	80.60 ± 25.31
Sexual enjoyment (BRSEE)	77.33 ± 34.53
Future perspective (BRFU)	53.15 ± 34.60
Symptom scales/items	
Systemic therapy side effects (BRST)	26.65 ± 20.35
Breast symptoms (BRBS)	18.24 ± 21.59
Arm symptoms (BRAS)	18.09 ± 23.08
Upset by hair loss (BRHL)	20.54 ± 35.14

**Table 5:** EORTC QLQ-C30 scales and correlations between the different dimensions of the subscale and S/A, T/A (STAI) and depressive symptoms (BDI).

EORTC QLQ-C30	S/A		T/A		BDI	
	r	*p*	r	*p*	r	*p*
Global health status/QoL	−0.386	0.000	−0.333	0.000	−0.546	0.000
Physical functioning (PF)	−0.291	0.000	−0.279	0.000	−0.561	0.000
Role functioning (RF)	−0.358	0.000	−0.361	0.000	−0.540	0.000
Emotional functioning (EF)	−0.590	0.000	−0.566	0.000	−0.616	0.000
Cognitive functioning (CF)	−0.415	0.000	−0.355	0.000	−0.539	0.000
Social functioning (SF)	−0.296	0.000	−0.317	0.000	−0.476	0.000
Fatigue (FA)	0.471	0.000	0.457	0.000	0.641	0.000
Nausea and vomiting (NV)	−0.008	0.907	0.014	0.841	0.200	0.005
Pain (PA)	0.286	0.000	0.347	0.000	0.560	0.000
Dyspnea (DY)	0.090	0.206	0.161	0.022	0.356	0.000
Insomnia (SL)	0.434	0.000	0.446	0.000	0.440	0.000
Appetite loss (AP)	0.244	0.000	0.264	0.000	0.448	0.000
Constipation (CO)	0.176	0.012	0.265	0.000	0.359	0.000
Diarrhea (DI)	0.085	0.229	0.203	0.004	0.253	0.000
Financial difficulties (FI)	0.245	0.000	0.283	0.000	0.336	0.000

**Table 6:** EORTC QLQ-BR23 scales and correlations between the different dimensions of the subscale and S/A, T/A (STAI) and depressive symptoms (BDI).

EORTC QLQ-BR23	S/A		T/A		BDI	
	r	*p*	r	*p*	r	*p*
Body image (BRBI)	−0.390	0.000	−0.396	0.000	−0.583	0.000
Sexual functioning (BRSEF)	−0.097	0.171	0.022	0.759	0.006	0.935
Sexual enjoyment (BRSEE)	−0.022	0.753	0.045	0.525	0.047	0.508
Future perspective (BRFU)	−0.385	0.000	−0.453	0.000	−0.327	0.000
Systemic therapy side effects (BRST)	0.248	0.000	0.403	0.000	0.563	0.000
Breast symptoms (BRBS)	0.297	0.000	0.162	0.022	0.314	0.000
Arm symptoms (BRAS)	0.270	0.000	0.180	0.011	0.359	0.000
Upset by hair loss (BRHL)	0.227	0.000	0.163	0.021	0.242	0.001

## Data Availability

The data underlying this article cannot be shared publicly to maintain the privacy of individuals that participated in the study. The data will be shared on reasonable request to the corresponding author.
